# Apropos: ‘Preliminary evaluation on the efficiency of the kit Platelia Dengue NS1 Ag-ELISA to detect dengue virus in dried *Aedes aegypti*: a potential tool to improve dengue surveillance’

**DOI:** 10.1186/1756-3305-7-205

**Published:** 2014-04-29

**Authors:** Subhash C Arya, Nirmala Agarwal

**Affiliations:** 1 Sant Parmanand Hospital, 18 Alipore Road, Delhi 110054, India

**Keywords:** Dengue, NS1 antigen, ELISA kit, Surveillance, Point-of-care tests

## Abstract

Only simple, point-of-care, assay formats of the Platellia Dengue NS1 Ag-ELISA would be suitable to identify Dengue virus in *Aedes aegypti* mosquitoes in dengue-endemic areas lacking sophisticated laboratory infrastructure and trained laboratory personnel.

## To the Editor

We compliment the team of investigators from Brazil for their meticulous evaluation of the Platellia Dengue NS1 Ag-ELISA assay to detect dengue virus (DENV) in dried *Aedes aegypti* mosquitoes [1] because it would be extremely valuable for disease surveillance in communities. Nevertheless, for the maximum utility of the technique, it would be crucial to offer it in a simple, 1–2 step, point-of-care assay format.

The perspective format should not only be user friendly but should not rely on availability of well-equipped laboratory premises and trained laboratory personnel. In several urban areas and remote locations in DENV-endemic, resource-poor countries, laboratory infrastructure is poor and private clinical laboratories might be the only ones serving a vast population. The inadequate building capacity, pathology and laboratory methods training, supply of reagents and maintenance of existing equipment have been far from ideal [2].

The utility of a rapid-point-of care laboratory diagnostic employing a commercial NS1 Ag STRIP (Biorad-Laboratory, Maines-la-Courquette, France) were encouraging in Taiwan. The Dengue NS1 Ag STRIP was a useful tool for an early dengue diagnosis. Its use increased the diagnostic sensitivity and decreases the need of examination of convalescent samples [3]. Furthermore, commercial availability of the point-of-care assay kits like the Dengue Duo, the one-step NS1 Ag and IgM/IgG test (Standard Diagnostic Inc., Ingbert, Germany) has been an asset during disease outbreaks. Its utility was immense among the patients presenting with different phases of primary or secondary dengue infection at a private hospital located in Delhi, India during the 2010 outbreak [4].

Last but not least, basic studies should also be undertaken with dried *Aedes aegypti* mosquitoes [1] to establish the utility of the future sensor based technology to diagnose DENV in mosquitoes. Employing an all-optical fiber sensor based on Localized Surface Plasmon Resonance (LSPR) and specular reflection from gold nanoparticles (AuNPs), it was able to detect NS1 antigen at different concentrations, with a limit of quantification estimated to be 0.074 μg/ml = 1.54 nM. The performance sensor for DENV diagnosis in the acute phase of the infection was encouraging [5].

## Competing interests

The authors declare that they have no competing interests.

## Authors’ contributions

SCA: Collected the references and informal discussion with clinicians. NA: Drafted the manuscript including editing of the text. Both authors read and approved the final manuscript.

## References

[B1] Sylvestre G Gandini M de Araújo JM Kubelka CF Lourenço-de-Oliveira R Maciel-de-Freitas R Preliminary evaluation on the efficiency of the kit Platelia Dengue NS1 Ag-ELISA to detect dengue virus in dried Aedes aegypti: a potential tool to improve dengue surveillance Parasit Vectors 2014 7 1 155 10.1186/1756-3305-7-155 24690324 PMC3998069

[B2] Amukela TK Michael K Hanes M Miller RE Brooks Jackson J External quality assurance performance on clinical research laboratories in Sub-Saharan Africa Am J Clin Path 2012 138 720 723 10.1309/AJCP8PCM4JVLEEQR 23086773

[B3] Huang C-H Kuo L-L Yang KD Lin P-S Po-Liang L Lin C-C Chang K Chen T-C Lin W-R Lin C-Y Chen Y-H Ho-Sheng W Laboratory diagnostics of dengue fever: An emphasis on the role of commercial dengue virus nonstructural protein 1 antigen rapid test J Microbiol Immunol Infect 2013 46 358 365 10.1016/j.jmii.2012.07.011 23041057

[B4] Arya SC Agarwal N Parikh SC Agarwal S Simultaneous detection of dengue NS1 antigen, IgM plus IgG and platelet enumeration during an outbreak Sultan Qaboos Univer Med J 2011 11 470 476 PMC3206749 22087395

[B5] Camara AR Gouvêa PM Dias AC Braga AM Dutra RF de Araujo RE Carvalho IC Dengue immunoassay with an LSPR fiber optic sensor Opt Express 2013 21 22 27023 27031 10.1364/OE.21.027023 24216926

